# Editorial: Community series in gut microbiota and immunity in health and disease: dysbiosis and eubiosis’s effects on the human body, volume II

**DOI:** 10.3389/fimmu.2026.1802846

**Published:** 2026-02-13

**Authors:** Sandip K. Wagh, Servaas Antoine Morre, Veronica I. Dodero

**Affiliations:** 1 Department of Biological Sciences, School of Science, Sandip University, Nashik, India; 2 Department Genetics and Cell Biology, Institute for Public Health Genomics, Research Institute GROW, FHML, Maastricht University, Maastricht, Netherlands; 3 Faculty of Chemistry, Bielefeld University, Bielefeld, Germany

**Keywords:** *Akkermansia muciniphila* (AKK), chronic kidney disease - mineral and bone disease (CKD-MBD), dermatitis atopic, dysbiosis, eubiosis, gut-liver axis, idiopathic short stature (ISS)

This Research Topic represents the second volume of the Community Series on *Gut Microbiota and Immunity in Health and Disease: Dysbiosis and Eubiosis’s Effects on the Human Body.* It builds on growing evidence that host–microbiota–environment interactions are central to nutritional and human health and disease (1, 2). From early-life microbial exposure through adulthood, the gut microbiota plays a pivotal role in maintaining metabolic homeostasis and immune competence (eubiosis). In contrast, microbial imbalance (dysbiosis) is associated with a broad spectrum of chronic conditions, including metabolic, autoimmune, cardiovascular, renal, neurological, and mental health disorders. Environmental factors, including diet, lifestyle, and genetics, critically shape these microbial ecosystems. This underscores the importance of integrated, systems-level approaches. We are proud to report that the five articles in this volume, contributed by 26 authors, collectively address key aspects of gut microbiota–immune–metabolic interactions. Three original research articles demonstrate how lifestyle and diet modulate microbial composition and clinical outcomes. Xu et al. reported an integrative animal–human study linking dietary patterns, microbial shifts, and immune responses to atopic dermatitis. Zeng et al. presented a pediatric study showing that diet, exercise, and sleep are associated with distinct gut microbial profiles in children with idiopathic short stature. Wang et al. reported a cross-sectional analysis of 2,947 patients with chronic kidney disease using NHANES data. Their findings indicate that microbiota-friendly diets are associated with reduced hypoalbuminemia in chronic kidney disease, underscoring the importance of dietary quality over quantity. Complementing these original research articles, two reviews were accepted. Gao et al. conducted a systematic bibliometric analysis to examine global research trends on Akkermansia muciniphila, a probiotic associated with IBD, obesity, and T2DM, over the past decade. The review characterizes this probiotic’s scientific development and health-related associations. Finally, a narrative review by Rao et al. highlights the intestinal microbiota as a key regulator of drug metabolism and therapeutic response via the gut–liver axis.

This editorial outlines key conclusions from the five featured articles. First, Xu et al. linked dietary patterns to changes in gut microbiota and their role in atopic dermatitis (AD). Their murine and human studies showed that AD is characterized by altered microbial diversity and composition, alongside dietary differences, suggesting a diet–microbiota–immune axis in AD. Results support the idea that plant-rich, low–refined-grain diets may remodel the gut microbiota in ways relevant to disease, a finding that merits further validation.

Zeng et al. profiled the gut microbiota in 58 children with idiopathic short stature and examined links to lifestyle factors. Microbial species differed in short-stature severity, with dietary diversity—particularly legume intake—associated with greater microbial diversity. Lifestyle factors, such as diet, exercise, and sleep, were closely associated with differences in these microbial communities between groups.

Wang et al. analyzed data from 2,947 chronic kidney disease (CKD) patients and found that adherence to a microbiota-friendly diet was associated with higher serum albumin and lower risk of hypoalbuminemia, regardless of total energy or protein intake. High-quality diets rich in fiber and whole grains were key, suggesting the importance of dietary composition for nutritional status in CKD.

Gao et al. surveyed global research on the probiotic Akkermansia muciniphila, identifying field growth, major contributors, and central themes, including links to metabolic and inflammatory diseases. These findings reinforce the prominence of probiotics in microbiome research and the need for further clinical studies.

Rao et al. reviewed how the intestinal microbiota influences drug metabolism and therapeutic response, both directly and indirectly through its effects on liver immune function. Multiple immune cell types mediate this regulation, impacting treatment outcomes.

The review concludes that targeting gut microbiota diversity could improve drug safety and efficacy, with approaches such as phage therapy showing promise but needing further validation. Epigenetic factors also contribute to variability in drug responses, underscoring the need for integrated research to advance precision medicine for liver and inflammatory diseases.

Collectively, this Research Topic emphasizes that gut microbiota composition, immune regulation, and environmental exposures are tightly interconnected determinants of health and disease. The findings support microbiota-targeted nutritional and therapeutic strategies and underscore the need for microbiome- and immune-informed precision medicine approaches to improve prevention, diagnosis, and treatment across diverse disease contexts.

## References

[1] Tan J Navarro S Macia L . Editorial: Deciphering host-gut microbiota communication in immunity and disease. Front Nutr. (2023) 10:1178039. doi: 10.3389/fnut.2023.1178039, PMID: 37051121 PMC10083485

[2] Dodero VI Morre SA Behzadi P . Editorial: Gut microbiota and immunity in health and disease: dysbiosis and eubiosis's effects on the human body. Front. Immunol. (2024) 15:1536258. doi: 10.3389/fimmu.2024.1536258, PMID: 39742257 PMC11685867

